# Virtual Reality in the Field of Gastroenterology: Entering a New Era

**DOI:** 10.14309/crj.0000000000002109

**Published:** 2026-04-24

**Authors:** Hema Sameera Pinnam, Bhanu Siva Mohan Pinnam, Harshita Malik, Hareesha Rishab Bharadwaj, Hassam Ali, Dushyant Singh Dahiya

**Affiliations:** 1 Department of Internal Medicine, Jagadguru Sri Shivarathreeshwara Medical College, Mysuru, Karnataka, India; 2 Division of Gastroenterology, Hepatology & Motility, The University of Kansas School of Medicine, Kansas City, KS; 3 Department of Internal Medicine, Kasturba Medical College, Manipal, Karnataka, India; 4 Department of Internal Medicine, Royal Stoke University Hospital, University Hospitals of North Midlands NHS, Stoke-on-Trent, UK; 5 Department of Gastroenterology, Hepatology and Nutrition, East Carolina University/Brody School of Medicine, Greenville, NC

**Keywords:** gastroenterology, hepatology, training, medical education, virtual reality

## INTRODUCTION

Medical education has undergone rapid evolution in recent years with the integration of artificial intelligence (AI) and simulation-based learning. In gastroenterology, virtual reality (VR) training offers a promising method for improving procedural skills and, ultimately, patient outcomes. However, our enthusiasm for VR has often outpaced the evidence on its most efficient use, risking investment in expensive technology without defining competency benchmarks or structured curricula. Although it was initially only explored for training purposes, our current body of evidence is extending into applying VR in therapeutic interventions such as periprocedural pain management. Moreover, the use of other AI-based tools, such as augmented reality (AR) and mixed reality (MR), is increasingly being studied. Although VR involves viewing a simulated three-dimensional environment through a headset, AR overlays simulated visuals onto a live-environment image.^1^ MR, on the other hand, is a mix of VR and AR, which allows manipulation of both virtual and real elements, allowing a physician to overlay virtual images on a patient's anatomy intraprocedurally and also create holographic models of patient anatomy.^2^ As research in this field continues to grow, we must move closer to creating a structured and well-balanced approach to using VR. Gastroenterology training is at a crossroads. AI technology is advancing rapidly, yet most fellowship programs lack a deliberate strategy for integrating it. If we do not define how VR should be used, its adoption will be limited and pushed by availability rather than educational or clinical value.

## VR IN GASTROENTEROLOGY TRAINING

Simulation has become an important part of gastroenterology training, but its role remains uneven across different procedures. Although simulators exist for many endoscopic techniques, most of the evidence today exists around esophagogastroduodenoscopy and colonoscopy. For more complex procedures such as endoscopic ultrasound and endoscopic retrograde cholangiopancreatography (ERCP), creating realistic VR simulations remains challenging. Still, it would be a mistake to underestimate how quickly this technology has evolved. Dismissing VR as a crude tool is no longer defensible, as newer models have advanced rapidly. There has been a huge evolution in training technology since the first plastic experimental model in 1974. Today, we have advanced systems such as animal-based and computer-based simulators. The Erlangen Endo-Trainer and compact-Erlanger Active Simulator for Interventional Endoscopy are animal-based simulators that use porcine specimens to practice skills such as ulcer hemostasis and variceal treatment. On the other hand, rapid evolution has occurred in computer-based simulators such as the AccuTouch Endoscopy Simulator by Immersion Medical Corp and the GI-Mentor II model by Simbionix Corp; these systems also include didactic training modules and progressively challenging cases for practice.^3^ Early systems like the GI Mentor offered relatively basic anatomical replication with force feedback.^4^ However, newer platforms now simulate a broad range of colorectal pathologies and provide a much more realistic procedural experience.^5^ These simulators show us the breadth of technologies currently available for procedural training.

What is increasingly clear is that VR training has real value, particularly early in training. Whether it outperforms conventional patient-based learning is no longer the question we should be asking; instead, we should emphasize using VR-based training for novices to fill an early skill gap. It allows trainees to build fundamental skills in a low-stakes environment, without the pressure or ethical concerns of learning on real patients. This also saves time for trainees and supervisors by shifting early mistakes away from the clinical setting. An important educational advantage of VR simulation is the ability to provide real-time feedback and expert guidance, including remote input from experienced endoscopists globally. This will broaden the trainees' exposure to different procedural techniques and perspectives. VR training also allows for the practice of key nontechnical skills. Communication, teamwork, and decision making during emergencies are hard to teach in real time. However, simulated scenarios can allow trainees to practice responses to high-stress situations and mentally prepare for the realities of endoscopy.

Although we acknowledge that cost and access are major barriers, programs need to prioritize investing in training infrastructure; with the rapid evolution of technology and AI, they must decide whether to lead this transformation or catch up later. Several studies have demonstrated that learning curves between simulation-based and conventional training tend to converge at higher levels of procedural difficulty. Trainees who underwent VR training performed significantly better than those with no training, demonstrating higher independent completion rates, improved mucosal visualization, and superior overall performance ratings. However, conventionally trained trainees exhibited higher rates of independent procedure completion.^6^ This must not be viewed as a limiting factor of VR. In fact, it highlights its value in gaining early skills. Cultivating foundational skills such as hand-eye coordination, torque control, and scope navigation is now made possible in a safe, reproducible environment. Previous trials have shown that structured learning with components such as didactic teaching, expert feedback, and increasing technical difficulty progressively yielded greater performance improvement than self-regulated learning without a clear direction.^7,8^

The generalizability of these studies remains limited, as conducting large-scale meta-analyses is currently challenging because of variation in assessment tools across studies. More recent syntheses of literature make one point very clear: VR should not be questioned as a wholesale alternative to conventional training.^9^ Its advantages are best used in a procedure-specific and stage-dependent manner; most benefits are seen in early skill acquisition and simpler procedures. The lack of clear gains in complex procedures emphasizes the limits of simulation technology and the importance of clinical judgement and procedural nuance.^10^ Using VR deliberately for introductory training without extending its role into areas where conventional training remains irreplaceable is key to ensuring appropriate use of VR in teaching. These findings underscore the need to identify which procedures are most amenable to VR-based education. At present, VR may be best suited for introductory training in esophagogastroduodenoscopy and colonoscopy, rather than highly complex procedures such as ERCP. The inconsistency in VR's effectiveness, especially in advanced procedures, raises critical questions for programs. Should VR be integrated at the beginning of training, where it is most effective, and then replaced with real-patient experience as trainees advance?

In summary, VR-based training has the potential to significantly enhance procedural education in gastroenterology, particularly for novice learners. Future efforts should focus on how best to integrate VR with traditional teaching methods and develop structured, evidence-based curricula that maximize learning outcomes.

## AI BEYOND VR—AR AND MR

AI in gastroenterology is now moving beyond VR and into newer forms of simulation technology. AR and MR, although still early in development, have the potential to change how endoscopic procedures are performed and taught.

In practice, AR works by overlaying computer-generated information onto the live endoscopic image. This added layer can draw attention to subtle mucosal abnormalities and may help endoscopists detect polyps and early tumors more quickly and accurately. Computer-aided detection is an AI-based system that uses algorithms to detect mucosal lesions and an AR overlay to identify polyps.^11^ Literature remains varied at the moment, with some studies demonstrating significant benefit with computer-aided detection systems, and others showing no improvement.^12,13^ This inconsistency highlights an important limitation of the current literature. This is less likely a reflection of the technology itself but more an indication that higher quality and more standardized research is still needed before we can draw firm conclusions about its clinical application.

MR represents another promising direction, particularly from an educational perspective. MR systems use head-mounted displays to form three-dimensional holographic models that can be viewed and manipulated in real time. This approach may be especially useful for complex procedures such as ERCP, where two-dimensional fluoroscopic images can make it difficult for trainees to fully appreciate ductal anatomy.

An example of this application was reported by Tanisaka et al in Japan. In a case involving resectable hilar bile duct cancer, a 3-D model was created from computed tomography images to illustrate the patient's biliary anatomy. Trainees were taught using this model before the procedure, allowing experts to elaborate on the anatomical difficulties. During ERCP, fluoroscopic images were then interpreted alongside the previously generated 3-D model, which appeared to help trainees better understand the spatial relationships within the biliary tree.^14^ Although this represents an early stage of application, it shows us how MR could bridge a learning gap in anatomical difficulty.

Taken together, augmented and MR technologies point toward a future in which simulation and real-time guidance are increasingly integrated into gastrointestinal practice. As with VR, the challenge will not be whether these tools work on their own, but how they are incorporated into training and clinical practice.

## INTRAPROCEDURAL PAIN MANAGEMENT

Pain management during endoscopy and colonoscopy has traditionally relied on analgesia and sedation. Although general anesthesia and opioid-based regimens are effective, they are not without risk and can be associated with complications during or after the procedure. For this reason, there has been growing interest in alternative approaches that could reduce discomfort while minimizing medication use. VR has been explored as a distraction-based tool for pain control in minimally sedated or unsedated patients, primarily in upper GI endoscopies and colonoscopies. Immersive VR engulfs the patient's attention and reduces pain by engaging them with auditory and visual stimulation; neurophysiologically, this may activate descending inhibitory pathways to reduce pain transmission at the spinal level as per the Gate Control Theory.^15^ By shifting attention away from the procedure, VR may reduce the subjective experience of pain and anxiety. However, results from existing studies have been mixed. Some trials have reported meaningful reductions in perceived pain and improved patient tolerance, whereas others have found no significant benefit compared with no pain medication or sedation.^16,17^

These mixed findings suggest that the role of VR in procedural pain control is still evolving. VR may be helpful for selected patients or specific procedures, rather than as a universal aid. Although VR continues to show promise as both a diagnostic and therapeutic adjunct in gastroenterology, more rigorous and standardized research will be needed before its effectiveness in pain management can be clearly defined.

## FUTURE IMPLICATIONS

As medical research continues to move at an unprecedented pace, new technologies and AI-assisted tools are increasingly shaping the direction of modern medicine. In gastroenterology, the procedural side of the specialty is both highly rewarding and technically demanding. Developing the skills needed to perform these procedures safely and effectively takes years of training, along with significant time and resource investment from both physicians and hospitals.

In this context, VR has the potential to play an important supporting role. If used thoughtfully, it can help trainees acquire foundational skills more efficiently and may even extend into therapeutic applications. This kind of support is not about speeding through training, but about making early learning safer, more structured, and more deliberate.

At the same time, it is important to remain realistic. As impressive as these technologies are, they cannot replace clinical judgement, experience, or human reasoning. So how can we integrate them with conventional training? Deciding when and where VR adds meaningful value will be key to ensuring that gastroenterology continues to evolve while maintaining high standards of patient care. Some methods to ensure efficient integration would be to use predefined metrics to assess trainees' performance, such as cecal intubation time and mucosal visualization, and to use proficiency-based training to advance trainees only once performance standards are met, rather than on passive participation. Using specific didactic modules, live performance feedback, and increasing the difficulty of procedures and anatomical complexity can further optimize VR use. Moreover, integrating VR-based performance into formal milestone evaluations and benchmarks for trainees will ensure meaningful engagement with simulator training. As gastroenterology programs adopt VR into training practices, they must ensure a structured approach. Incorporating competency frameworks, using multimodal approaches, and creating a practical curriculum will be paramount in ensuring that these technologies are used efficiently and appropriately. By proactively shaping the application of these technologies, programs can ensure that they are used meaningfully to enhance education and that innovation strengthens the quality of procedural training.

## DISCLOSURES

Author contributions: HS Pinnam, BSM Pinnam, and DS Dahiya made substantial contributions to the conception or design of the work or the acquisition, analysis, or interpretation of data for the work. All authors drafted the work and reviewed it for important intellectual content, agreed to be accountable for all aspects of the work in ensuring that questions related to the accuracy or integrity of any part of the work are appropriately investigated and resolved, and gave final approval of the version to be submitted. DS Dahiya is the article guarantor.

Financial disclosure: None to report.
